# Ultrasound-guided continuous parasacral ischial plane block for diabetic foot ulcer healing: study protocol for a single-centre, prospective, randomised, open-label, blinded-endpoint controlled trial

**DOI:** 10.3389/fendo.2026.1884667

**Published:** 2026-08-17

**Authors:** Peng Ye, Xuan Pan, Yongxin Huang, Jing Zhuang, Hanliang Fan, Yuqi Lin, Xinyi Yang, Danfeng Wang, Xiaochun Zheng, Chunying Zheng, Ting Zheng

**Affiliations:** 1 Department of Anaesthesiology, Shengli Clinical Medical College of Fujian Medical University, Fujian Provincial Hospital, Fuzhou University Affiliated Provincial Hospital, Fuzhou, China; 2 Fujian Provincial Key Laboratory of Emergency Medicine, Fujian Provincial Key Laboratory of Critical Care Medicine, Fujian Provincial Co-Constructed Laboratory of “Belt and Road,” Fujian Emergency Medical Centre, Fuzhou, China; 3 Department of Plastic and Burn, Shengli Clinical Medical College of Fujian Medical University, Fujian Provincial Hospital, Fuzhou University Affiliated Provincial Hospital, Fuzhou, China

**Keywords:** diabetic foot ulcer, parasacral ischial plane block, regional anaesthesia, sympathetic blockade, wound healing

## Abstract

**Introduction:**

Diabetic foot ulcers (DFU) represent a significant global health burden, drastically reducing quality of life and increasing amputation risk. Associated pain and peripheral vascular disease complicate management and healing. Continuous parasacral ischial plane block (CPIPB), a regional anaesthesia technique targeting the sacral plexus, has shown potential in preclinical studies and feasibility assessments to improve lower limb analgesia and perfusion by modulating sympathetic tone. This trial aims to evaluate the efficacy and safety of CPIPB combined with standard of care (SOC) compared to SOC alone for promoting the healing of chronic DFU.

**Methods and analysis:**

This study utilizes a single-centre, prospective, randomised, open-label, blinded-endpoint (PROBE) parallel-group design. A total of 78 participants aged 20–89 years with a DFU (University of Texas Classification 1A-2D) present for >4 weeks but <1 year, with an area between 1–20 cm², and adequate perfusion (ABI >0.7 or equivalent) will be recruited after a 2-week SOC run-in period (inclusion requires <30% wound area reduction). Participants will be randomised (1:1) using variable block randomisation to either: 1) CPIPB + SOC: ultrasound-guided catheter placement in the parasacral ischial plane for continuous infusion of 0.2% ropivacaine at 5 mL/h for 14 days, combined with SOC; or 2) SOC alone: including glycaemic control, appropriate debridement, and standard dressings. The primary outcome is the incidence of complete ulcer healing at 12 weeks. Secondary outcomes include time to complete healing, percentage change in ulcer area, proportion achieving ≥50% area reduction, ulcer recurrence, infection rates, pain scores (VAS), lower limb perfusion assessments (TcPO2, Peak Systolic Velocity), health-related quality of life (Wound-QoL), and safety events. Statistical analysis will primarily use the intention-to-treat principle, comparing groups using chi-square tests, t-tests/Mann-Whitney U tests, Kaplan-Meier analysis, and logistic/linear regression models adjusting for baseline covariates where appropriate.

**Discussion:**

This study aims to evaluate the effects of a 12-week treatment with CPIPB and SOC on patients with DFU. We hypothesize that, compared to SOC alone, CPIPB combined with SOC would significantly improve ulcer healing without provoking significant adverse reactions. These findings potentially pave the way for a safe and effective adjunctive therapy for DFU.

**Clinical trial registration:**

https://www.chictr.org.cn/, identifier ChiCTR2400083504.

## Introduction

Chronic non-healing ulcers, particularly diabetic foot ulcers (DFU), pose a significant global health challenge. It is estimated that up to 15% of individuals with diabetes mellitus will develop a lower extremity ulcer during their lifetime, leading to a significant impairment of quality of life and a dramatic increase in the risk of lower limb amputation (1). DFU are among the most common and severe complications of diabetes, arising from a complex interplay of factors including peripheral neuropathy, peripheral artery disease (PAD), and infection (2). Effective DFU management necessitates comprehensive strategies aimed at promoting ulcer healing and reducing amputation risk (3).

Pain is a frequent symptom for patients with DFU, primarily stemming from diabetic peripheral neuropathy (DPN) and PAD (4). DPN, encompassing sensory, motor, and autonomic nerve damage, is a critical risk factor for ulceration due to loss of protective sensation (5, 6). Concurrently, PAD, characterized by reduced lower limb blood flow, tissue hypoxia, and insufficient nutrient supply, can cause claudication, rest pain, and impaired wound healing, potentially leading to critical limb ischaemia, ulceration, and gangrene (7). Lower limb pain profoundly affects patients’ mobility, increases fall risk, hinders wound healing, and contributes to psychological comorbidities such as anxiety and depression (8). Furthermore, pain is an independent predictor of amputation risk in DFU patients (9, 10). Therefore, pain management is an important component of the treatment process for patients with diabetic foot ulcers. It requires a comprehensive consideration of factors such as neuropathy, vascular disease, and infection, and appropriate therapeutic measures should be taken to alleviate pain, improve the patient’s quality of life, and reduce the risk of amputation (11, 12).

PAD, especially vascular stenosis, significantly influences DFU treatment and prognosis (13). Chronic hyperglycaemia damages vessel walls, leading to stenosis or occlusion and subsequent ischaemia, increasing ulcer risk and impairing healing (14). This is often characterized by diffuse, multi-vessel, infra-popliteal disease with long occlusions, poor collateralization, medial calcinosis, and vasoconstriction, complicating revascularization efforts and wound care (15, 16). Addressing lower limb ischaemia is thus a cornerstone of DFU management.

Regional anaesthesia techniques targeting the lower limb may offer benefits beyond analgesia by modulating sympathetic tone and improving perfusion. Ultrasound-guided continuous parasacral ischial plane block (CPIPB) is a novel technique for continuous sacral plexus blockade. Our prior research standardized the CPIPB catheter placement procedure, confirming its feasibility and safety (17). We observed that CPIPB provides effective lower limb analgesia and appears to improve circulation via vasodilation, likely through blockade of sacral plexus and sacral sympathetic nerves (18). Subsequent preclinical work suggested that CPIPB promotes wound angiogenesis following sympathetic blockade, thereby accelerating DFU healing (19).

Given these preliminary findings, this clinical trial aims to determine whether CPIPB, as an adjunct to standard of care (SOC), can clinically accelerate the healing of chronic, non-healing DFU compared to SOC alone. We will conduct a randomised parallel-controlled trial to determine the impact of CPIPB on patients with DFU. We hypothesize that CPIPB would significantly improve ulcer healing without provoking significant adverse reactions compared to SOC. In addition, these findings potentially pave the way for a safe and effective adjunctive therapy for DFU.

## Methods and analysis

### Study design

**Figure 1:**
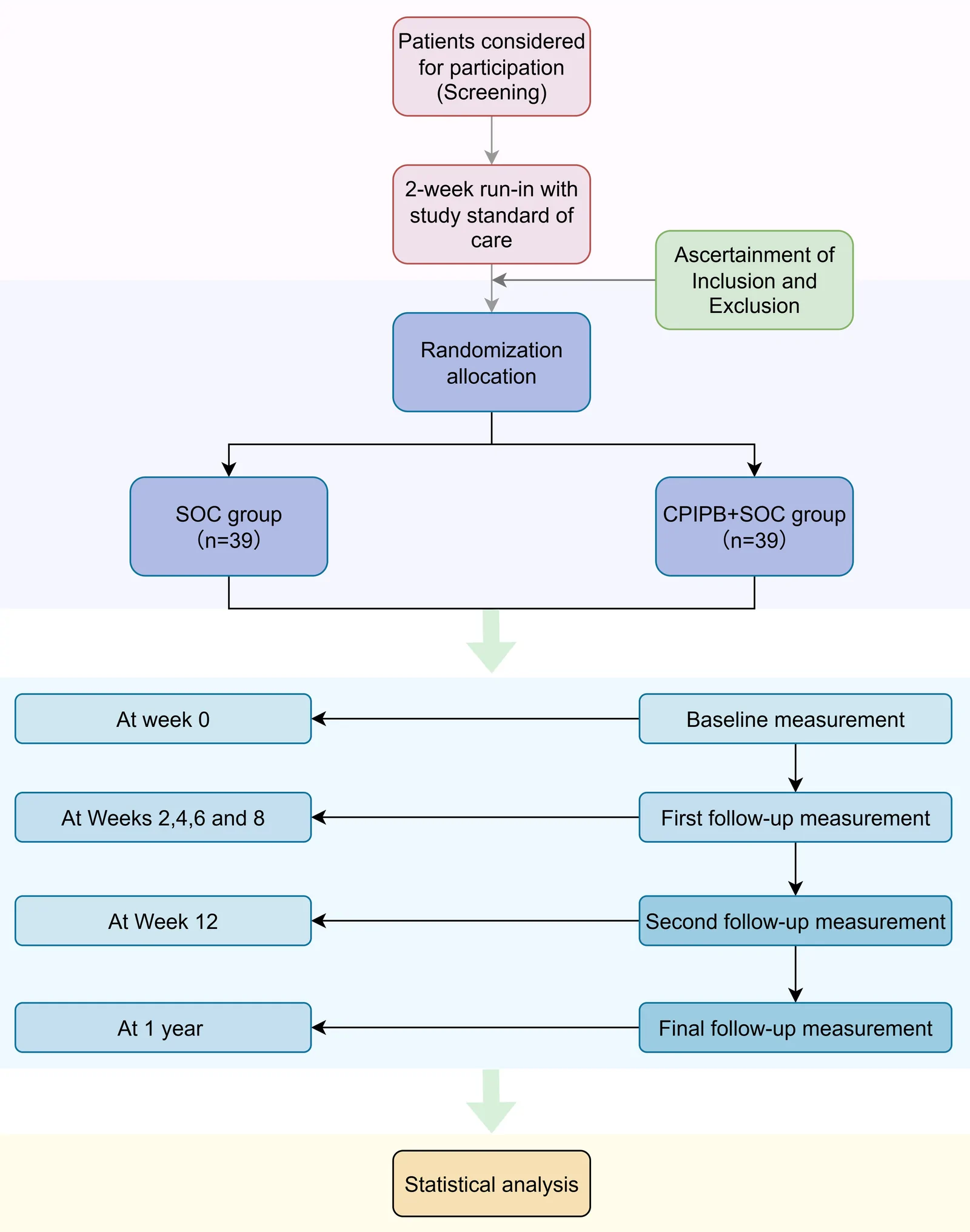
Trial flow chart.

This is a single-centre, prospective, randomised, open-label, blinded-endpoint (PROBE), parallel-group, controlled superiority trial. The PROBE design was specifically chosen to overcome the practical limitations of blinding patients and clinicians to the regional anaesthesia intervention, while ensuring rigorous, unbiased evaluation of the primary and secondary outcomes by blinded independent assessors. This study is conducted at Fuzhou University Affiliated Provincial Hospital in Fuzhou, China. This study will follow the Consolidated Standards of Reporting Trials (SPIRIT) guidelines (20). The study flowchart is shown in **Figure 1**.

### Study setting

The recruitment and follow-up will be conducted at the wound care outpatient clinics of Fuzhou University Affiliated Provincial Hospital by a multidisciplinary staff consisting of anaesthesiologist, wound care nurses, podiatrists and plastic surgeons. The staff will screen patients at the respective outpatient clinics according to the following eligibility criteria: Potential participants will undergo a 2-week run-in period receiving SOC. Those whose target ulcer area reduction is less than 30% after this period will be eligible for randomisation if they meet all inclusion and none of the exclusion criteria.

**Inclusion criteria:**

- Males and females aged between 20 and 89 years
- Documented diagnosis of type 1 or 2 diabetes
- Foot ulcer at or below ankle with duration >4 weeks to <1 year
  ■ If the index ulcer is post amputation, date of surgery must be >30days
  ■ If>1ulcer is present, largest is considered as the study index ulcer
  ■ Index ulcer must be ≥1cm from any other ulcers present on the foot
- Ulcer size ≥1 and ≤20 cm^2^ after debridement at start of run-in period
- Ulcer of UTC grade 1A, 1B, 1C, 1D, 2A, 2B, 2C, or 2D
- ABI >0.7 with a TcPO_2_,>30 mmHg, skin perfusion >30 mmHg, toe pressure >30 mmHg, or Duplex ultrasound with biphasic waveforms below the knee
- No planned revascularization procedure or vascular surgery within the last or next 30 days
- Subject and caregiver willing and able to comply with all specified care and visit requirements
- Subject has a reasonable expectation of completing the study
- Subject completed 2-week run-in period with <30% wound size reduction

**Exclusion criteria:**

- Evidence of gangrene on any part of affected limb
- Documented evidence of osteomyelitis on any part of affected limb
- Index ulcer has exposed bone
- Active Charcot foot on the study limb
- Uncontrolled diabetes: HbA_1c_>12% (108 mmol/mol)
- Renal dialysis or creatinine >2.5 mg/dL (221 μmol/L)
- Known immune insufficiency
- Active treatment for malignancy (not specific to study limb)
- Chronic steroid use or immunosuppressive agents within the last 3 months or anticipated to require them during the duration of the study
- Subject participated in another investigational device, drug, or biological trial within last 30 days
- Index ulcer exhibits signs of severe clinical infection that requires hospitalization or immediate surgical intervention
- Subject is pregnant at the time of screening
- Subject has had a deep vein thrombosis within the last 30 days
- Subject has received growth factor therapy, autologous platelet-rich plasma gel, bilayered cell therapy, dermal substitute, extracellular matrix, etc., within the screening period
- Local anaesthetic allergy

**Withdrawal criteria:**

- Occurrence of a serious adverse event (SAE) or complication rendering continuation inappropriate;
- Participant’s voluntary withdrawal (withdrawal of consent);
- Loss to follow-up;
- Significant protocol deviation impacting the ability to assess efficacy or safety; investigator’s decision that withdrawal is in the participant’s best interest.

### Handling of dropout cases

Participants who discontinue early or are lost to follow-up will be contacted for a final assessment whenever feasible. Every effort will be made to collect efficacy and safety data for these individuals. The reasons for withdrawal will be recorded in both the case report form and the trial conclusion form.

### Random allocation and blinding

#### Blinding

This is an open-label study. Participants, clinicians administering the intervention, and personnel assessing most clinical outcomes are aware of the treatment allocation. For specific objective measurements, such as ulcer area assessment, hemodynamic readings, or laboratory analyses, outcome assessors remain blinded, and statisticians conducting the final analysis also remain blinded until the analysis is complete.

### Assignment of interventions (randomisation and allocation)

Sequence Generation: A randomisation sequence will be generated using variable block sizes (2, 4, 6, or 8) with a 1:1 allocation ratio by a statistician independent of the clinical team, using R studio statistical software with a pre-specified random seed (20221023).

Allocation Concealment Mechanism: Due to the nature of the intervention, allocation concealment from the participants and the clinicians performing the intervention is not possible. However, the randomisation sequence will be managed by personnel not involved in patient recruitment or assessment to prevent foreknowledge of allocation before eligibility is confirmed.

Implementation: Once a participant completes the run-in period, meets all eligibility criteria, and provides final consent for randomisation, the study coordinator will contact the central randomisation service/personnel to obtain the treatment allocation according to the generated sequence.

#### Sample size

Based on previous research, the healing rate in the SOC group is approximately 47% (21). Our institutional pre-experiment (pilot study) of DFU patients receiving continuous regional block showed that approximately 80% of participants achieved complete wound closure within 12 weeks; thus, we hypothesized that CPIPB + SOC will increase the 12-week healing rate to approximately 80%. To detect this difference with 90% power (β=0.10) at a one-sided significance level of α=0.05, 35 participants are required per group. Accounting for an estimated 10% dropout rate, a total of 78 participants (39 per group) will be recruited.

#### Recruitment

Participants will be recruited from patients attending the DFU clinics. Potential participants meeting initial screening criteria will be approached by the study team, provided with information, and invited to provide informed consent for the run-in period and potential randomisation. Strategies to enhance recruitment include clinician education and regular screening logs.

### Quality control

To uphold the integrity of clinical trials, both sponsors and participants must adhere to established standard operating procedures (SOPs) for robust quality control and the effective implementation of the overarching quality assurance system. All observational data and emergent findings generated during the trial necessitate rigorous verification to substantiate data reliability. Furthermore, it is imperative that all conclusions drawn from clinical trial results are demonstrably rooted in and supported by the primary data. Continuous quality control mechanisms should be integrated throughout the entire data processing pipeline, ensuring that all recorded information is both dependable and accurately handled.

### Monitoring

The sponsor designates a monitor responsible for conducting routine on-site visits to the investigational site. These visits are essential for verifying strict adherence to all aspects of the study protocol and for cross-referencing original data against the Case Report Form (CRF) to confirm data consistency.

### Interventions

#### Intervention group (CPIPB + SOC)

Participants randomised to this group will receive SOC (detailed below) combined with the CPIPB procedure.

##### CPIPB procedure

The specific treatment process includes patients undergoing parasacral ischial plane catheterization by the same treatment team in accordance with the study method proposed by Ye et al. (17).

##### Infusion

Post-procedure, a continuous infusion of 0.2% ropivacaine is delivered at 5 mL/h via an infusion pump for 14 days. This specific regimen is chosen based on our previous clinical feasibility and dose-finding trials (17, 19); this concentration and rate selectively block sensory and sympathetic fibres while sparing motor function, thereby preserving patient mobility and enabling essential rehabilitation. Delivering 10 mg of ropivacaine per hour (5 mL/h) is also well below the established systemic toxic threshold (0.6 mg/kg/h) (22), ensuring long-term safety. The 14-day infusion duration is derived from our pilot data and preclinical evidence (19), which indicate that a minimum of 10 to 14 days of sustained regional sympathetic blockade is required to downregulate inflammatory mediators, initiate microvascular remodelling, and promote robust local angiogenesis.

##### Control group (SOC)

Participants randomised to this group will receive standard of care (SOC) alone. To minimize performance bias and ensure study reproducibility, the SOC is highly standardized and encompasses several key clinical dimensions. First, glycaemic management will be overseen by a consulting endocrinologist, targeting an HbA1c < 8.0%, fasting blood glucose < 7.0 mmol/L, and postprandial blood glucose < 10.0 mmol/L (23). Second, sharp debridement of hyperkeratotic margins and non-viable tissues will be performed weekly by a dedicated, trained wound care podiatrist or nurse as clinically indicated. Third, wound dressings will be selected based on wound characteristics, utilizing either standard plain Vaseline gauze as specified in the protocol, or standardized moisture-retentive dressings, applied and changed every 2 to 3 days, or daily if clinically necessary. Fourth, standardized offloading interventions will be tailored to the anatomical location of the ulcer and patient compliance, including the use of custom-molded therapeutic footwear, felt-on-foam pressure-relieving pads, or non-removable knee-high walkers (24). Fifth, local or systemic infections will be managed strictly according to standard protocols; systemic antibiotic therapy will only be initiated if the patient exhibits clear clinical signs of infection in accordance with the IWGDF/IDSA guidelines, whereas prophylactic antibiotics are strictly prohibited (25). Finally, participants will attend regular, scheduled follow-up visits for comprehensive and standardized wound assessment and care.

### Concomitant medications and care

All participants will continue necessary medications for comorbidities (e.g., diabetes, hypertension, dyslipidaemia). Use of systemic analgesics will be recorded. Both groups will receive education on DFU prevention and self-care.

### Outcomes

#### Primary outcome

The primary study end point was the percentage of ulcers in each group achieving 100% healing at 12 weeks. Complete healing is defined as 100% re-epithelialization without drainage or dressing requirements, confirmed at two consecutive visits 2 weeks apart by an independent, blinded wound specialist. To ensure objective evaluation, wound areas will be traced and verified using computerized planimetry (ImageJ) on standardized digital photographs (taken with fixed distance, positioning, and illumination). These photographs will be securely archived and cross-checked by a central, blinded adjudication committee of two senior plastic surgeons.

#### Secondary outcomes

Secondary end points included wound area reduction, 12-month incidence of both recurrence and complete healing, lower limb haemodynamic assessments, incidence of amputation,pain assessment, Wound-QoL assessment, and adverse events.

### Participant timeline

Participants will attend visits at baseline (week-2 and Week 0, randomisation), Week 2, Week 4, Week 6, Week 8, Week 12 and year 1. The CPIPB infusion runs for the first 14 days (Week 0 to Week 2). Data collection points for specific outcomes are outlined in the schedule of assessments (**Table 1**).

**Table 1:** Schedule for data collection, the process of the assessments per visit.

Measures	Baseline	Intervention phase					
Time(weeks)	-2-0	2	4	6	8	12	1 year
Informed consent	√						
Demographics and Past medical history	√						
Physical examination	√	√	√	√	√	√	√
Laboratory Tests	√	√				√	√
Hemodynamic Tests	√	√	√	√	√	√	√
Electrocardiogram	√						
Ascertainment of Inclusion and Exclusion Table	√						
Routine follow-up measurement table	√	√	√	√	√	√	√
Adverse events	√	√	√	√	√	√	√
Endpoint events		√	√	√	√	√	√
Treatment adherence assessment	√	√	√	√	√	√	√

Treatment adherence assessment includes compliance with CPIPB infusion (Weeks 0-2) and long-term standard of care (e.g., offloading, wound care).

#### Baseline data

Demographics, medical history (diabetes type/duration, comorbidities, medications), ulcer characteristics (duration, location, UTC grade, area),electrocardiogram, baseline pain (VAS), baseline haemodynamics (ABI, TcPO2, temperature, PSV), baseline Wound-QoL, laboratory tests.

#### Follow-up data

This includes ulcer healing status, ulcer area measurements, signs of infection, pain scores, hemodynamic measurements, Wound-QoL (assessed at 12 weeks), adverse events, block-related complications, factors related to ulcer recurrence, changes in concomitant medications and treatment adherence.

#### Methods

Clinical examination, validated questionnaires (VAS, Wound-QoL), specific devices for haemodynamic assessment (Doppler, TcPO2 monitor, thermometer), ulcer tracing/photography for area measurement, laboratory analyses, review of medical records. Data will be recorded on standardized Case Report Forms (CRFs), either paper-based or electronic.

### Statistical methods

The primary analysis will be conducted on the intention-to-treat (ITT) population, defined as all randomised participants regardless of adherence or withdrawal.

#### Primary outcome analysis

The proportion of participants achieving complete ulcer healing at 12 weeks will be compared between the CPIPB+SOC group and the SOC group using a chi-square test or Fisher’s exact test, as appropriate. Odds ratios (OR) with 95% confidence intervals (CI) will be calculated using logistic regression, potentially adjusting for important baseline covariates (e.g., baseline ulcer area, UTC grade, duration of the ulcer, and baseline perfusion status (TcPO2)). Furthermore, to explore potential variations in treatment response, prespecified subgroup analyses will be conducted based on baseline ulcer size, UTC grade, and baseline perfusion status.

#### Secondary outcome analysis

Time-to-event outcomes (time to healing, time to recurrence) will be analysed using Kaplan-Meier curves and compared using the log-rank test. Hazard ratios (HR) with 95% CIs will be estimated using Cox proportional hazards regression, adjusting for covariates.

Continuous outcomes (e.g., percentage area reduction, change in VAS, change in TcPO2, Wound-QoL scores) will be compared using independent t-tests or Mann-Whitney U tests, depending on data distribution. Analysis of covariance (ANCOVA) may be used to adjust for baseline values.

Binary secondary outcomes (e.g., proportion achieving ≥50% area reduction, infection incidence) will be compared using chi-square or Fisher’s exact tests, with ORs and 95% CIs from logistic regression.

Adverse event rates will be summarized descriptively and compared using Fisher’s exact test.

To address missing primary and secondary outcome data, multiple imputation by chained equations (MICE) will be performed as a sensitivity analysis under the missing-at-random (MAR) assumption. No interim analysis is planned for this study due to the relatively modest sample size (n=78). To control the family-wise error rate, the Bonferroni correction will be applied to adjust p-values for multiple comparisons among secondary outcomes. All statistical tests will be two-sided with a significance level set at P < 0.05. Statistical analysis will be performed using SPSS version 18.0 (SPSS, Inc., USA) and GraphPad Prism version 8.2.

### Data monitoring

Given the single-centre nature and the known safety profile of the interventions (SOC and regional anaesthesia with ropivacaine), a formal Data Monitoring Committee (DMC) may not be required, but safety will be closely monitored by the Principal Investigator and the local Ethics Committee. Regular reviews of recruitment, protocol adherence, and safety data will occur.

### Harms

Adverse events (AEs) and serious adverse events (SAEs) will be proactively monitored and recorded throughout the study. Daily clinical inspection of the catheter site will be conducted; if infection is suspected, the catheter will be removed immediately for tip culture, and empirical antibiotics initiated (26). To prevent Local Anaesthetic Systemic Toxicity (LAST), staff and patients will be educated on early symptoms, and 20% lipid emulsion will be kept readily available in the ward following ASRA guidelines (27). Catheters will be removed at protocol completion (Day 14) or earlier in the event of local infection, dislocation, severe pain, patient request, or suspected LAST. Participants will undergo routine ward surveillance and continuous vital-sign monitoring, with immediate unscheduled assessments triggered by any new symptoms. A 24-hour communication pathway is established between the clinical teams; in case of serious complications, the infusion will be stopped immediately and resuscitation initiated. All AEs will be assessed for severity and causality, and SAEs will be reported to the Ethics Committee within established timelines.

#### Hemodynamic safety monitoring and management

Vital signs will be monitored continuously during catheterization, every 4 hours for the first 24 hours in the ward, and twice daily at home. If significant hypotension (systolic blood pressure <90 mmHg or >20% reduction from baseline) or bradycardia (heart rate <50 bpm) occurs, the infusion will be paused, the research team will be notified, and standard resuscitation (fluids, ephedrine, or atropine) will be initiated. Concurrently, the patient will be evaluated for LAST; if suspected, the institutional lipid rescue protocol (20% lipid emulsion) will be activated immediately. In such cases, the intervention will be permanently discontinued, the catheter will be removed, and the participant will be withdrawn from the trial with a two-week safety follow-up.

## Discussion

This study protocol describes a randomised controlled trial designed to test the hypothesis that ultrasound-guided continuous parasacral ischial plane block (CPIPB), as an adjunct to standard of care (SOC), can significantly enhance the healing of chronic diabetic foot ulcers (DFUs). Our rationale is predicated on the understanding that the pathophysiology of non-healing DFUs is multifactorial, with chronic ischemia and debilitating pain representing two critical, interconnected barriers to successful wound closure. While SOC addresses factors like glycaemic control and infection, it often fails to adequately resolve the underlying microvascular dysfunction and neuropathic pain. Therefore, the proposed intervention, CPIPB, is being investigated as a means to actively modulate physiological responses, complementing SOC in addressing these core deficits.

A primary obstacle to DFU healing is the profound peripheral artery disease (PAD) that characterizes the diabetic limb, frequently manifesting as diffuse, infra-popliteal disease with significant medial calcinosis and poor collateralization (15, 28, 29). This creates a chronic state of tissue ischemia and a hypoxic wound microenvironment that is fundamentally hostile to the cellular processes of healing, including angiogenesis, granulation tissue formation, and re-epithelialization (30, 31). Compounding this structural vascular disease is a functional component of autonomic neuropathy, often characterized by sympathetic overactivity. This leads to exaggerated vasoconstriction, further compromising blood flow to the distal extremities and impeding the delivery of oxygen and nutrients essential for tissue repair (32).

CPIPB is uniquely positioned to counteract this ischemic state. By delivering a continuous local anaesthetic infusion to the sacral plexus, the technique is intended to produce a targeted and sustained blockade of the efferent sympathetic fibres that regulate vascular tone in the lower limb (18, 19). We hypothesize that this sympatholysis will induce vasodilation, thereby increasing perfusion in the microvasculature of the foot. This proposed mechanism is not merely theoretical; it provides a direct biological rationale for the anticipated improvements in wound healing. The planned secondary outcomes, specifically the measurement of transcutaneous oxygen pressure (TcPO2) and Peak Systolic Velocity (PSV), are designed to provide objective, quantitative evidence of this physiological effect. An increase in these parameters in the CPIPB group would strongly support the contention that the intervention enhances local tissue oxygenation, directly addressing a core element of DFU pathophysiology and creating a more permissive environment for wound closure (19).

Pain in patients with DFUs is a complex and debilitating symptom, arising from both diabetic peripheral neuropathy (DPN) and ischemic vascular disease (33, 34). This is not merely a quality-of-life issue; chronic pain can actively hinder the healing process through a maladaptive physiological feedback loop (35). Persistent pain stimuli can trigger a systemic stress response, including activation of the sympathetic nervous system, which in turn can exacerbate vasoconstriction, worsen tissue ischemia, and thus intensify the pain. This vicious cycle perpetuates a state of impaired perfusion and creates a physiological barrier to healing (36–38).

The CPIPB intervention is designed to interrupt this cycle at multiple points. First, by providing continuous somatic nerve blockade of the sacral plexus, it offers potent and sustained analgesia, directly addressing both the neuropathic and ischemic pain components. This is expected to significantly reduce Visual Analogue Scale (VAS) scores. Second, through the simultaneous sympathetic blockade discussed above, it alleviates the ischemia that often drives the pain (18, 19). By effectively managing pain, CPIPB may not only improve patient comfort and mobility but also mitigate the negative downstream effects of sympathetic activation, thereby fostering a physiological state more conducive to tissue regeneration. This dual therapeutic action—providing analgesia while concurrently improving perfusion—differentiates CPIPB from conventional systemic analgesics, which lack a direct effect on vascular hemodynamic.

While the primary endpoint of this trial is complete ulcer healing, a truly effective DFU therapy must also address the profound negative impact the condition has on a patient’s health-related quality of life (HRQoL). The constant pain, reduced mobility, and persistent threat of amputation contribute to significant psychological distress, including anxiety, fears and depression (35, 39, 40). The Wound-QoL questionnaire was specifically chosen as a secondary outcome to capture these broader, patient-centred dimensions of treatment success. We hypothesize that the benefits of CPIPB will extend far beyond wound metrics. The synergistic effects of superior pain relief and accelerated wound healing are expected to translate into clinically meaningful improvements in HRQoL. Effective pain control can restore sleep patterns and improve mobility, while visible progress in wound healing can provide a powerful psychological boost, reducing anxiety and restoring a sense of control. Therefore, the anticipated improvement in Wound-QoL scores would not be an incidental finding but rather a direct reflection of the intervention’s success in alleviating the comprehensive burden of DFU.

Should this trial demonstrate the superiority of CPIPB plus SOC over SOC alone, the clinical implications would be substantial. This technique could offer a novel, safe, and effective adjunctive therapy for a challenging patient population that currently has limited options. By targeting key pathophysiological drivers of non-healing ulcers, CPIPB could potentially accelerate healing times, reduce the incidence of complications like severe infections, and ultimately lower the rate of major amputations. The findings will also pave the way for future research. Larger, multicentre trials will be necessary to confirm our results and establish the generalizability of the technique. Subsequent studies could explore the optimization of the CPIPB protocol, including the ideal duration of infusion and concentration of local anaesthetic. Furthermore, investigating the efficacy of CPIPB in specific subgroups of DFU patients, such as those with severe ischemic pain who are not candidates for revascularization, would be a valuable next step. In conclusion, this study protocol outlines a trial for a mechanistically targeted therapy that has the potential to significantly improve clinical outcomes and quality of life for patients suffering from the devastating consequences of diabetic foot ulcers.

## Limitations

Several limitations of this study protocol should be acknowledged. First, this is a single-centre trial conducted at a provincial hospital, which may limit the external validity and generalizability of our findings to other healthcare settings with different patient demographics. Second, although we have implemented a rigorous PROBE design and blinded independent outcome assessors to minimize detection bias, the open-label nature of the intervention may still theoretically introduce some performance bias. Third, the 14-day continuous CPIPB infusion is relatively short compared to the 12-week wound healing observation window, though it is designed to cross the critical early window of microvascular recovery and angiogenesis. Finally, the inclusion requirement of a 2-week run-in period with <30% wound area reduction may slightly restrict the representativeness of our randomized cohort to the broader diabetic foot ulcer (DFU) population by excluding acute, highly responsive, or rapidly healing wounds. Nevertheless, this run-in design significantly enhances the internal validity of the trial by focusing specifically on hard-to-heal, truly chronic ulcers that are most in need of advanced adjunctive therapies, thereby ensuring a more stringent and clinically meaningful evaluation of the efficacy of CPIPB.
